# Whole-genome characterization of large-cell lung carcinoma: A comparative analysis based on the histological classification

**DOI:** 10.3389/fgene.2022.1070048

**Published:** 2023-01-04

**Authors:** Xiaowei Wu, Jin Yin, Yu Deng, Yukun Zu

**Affiliations:** ^1^ Department of Thoracic Surgery, Tongji Hospital, Tongji Medical College, Huazhong University of Science and Technology, Wuhan, China; ^2^ Departments of Hematology, Tongji Hospital, Tongji Medical Collage, Huazhong University of Science and Technology, Wuhan, China

**Keywords:** non-small cell lung cancer (NSCLC), Large-cell lung cancer (LCLC), large cell neuroendocrine carcinoma (LCNEC), whole-genome sequencing, histological classification

## Abstract

**Background:** According to the 2015 World Health Organization classification, large cell neuroendocrine carcinoma (LCNEC) was isolated from Large-cell lung cancer (LCLC) tumors, which constitutes 2%–3% of non-small cell lung cancer (NSCLC). However, LCLC tumors are still fairly vaguely defined at the molecular level compared to other subgroups.

**Materials and Methods:** In this study, whole-genome sequencing (WGS) was performed on 23 LCLC and 15 LCNEC tumor specimens. Meanwhile, data from the TCGA (586 LUADs and 511 LUSCs) and U Cologne (120 SCLCs) were analyzed and compared.

**Results:** The most common driver mutations were found in *TP53* (13/23, 57%), *FAM135B* (8/23, 35%) and *FAT3* (7/23, 30%) in LCLC, while their counterparts in LCNEC were *TP53* (13/15, 87%), *LRP1B* (6/15, 40%) and *FAT1* (6/15, 40%). Notably, *FAM135B* mutations only occurred in LCLC (*P* = 0.013). Cosmic signature analysis revealed widespread defective DNA mismatch repair and tobacco-induced mutations in both LCLC and LCNEC. Additionally, LCNEC had a higher incidence of chromosomal copy number variations (CNVs) and structural variations (SVs) compared with LCLC, although the differences were not statistically significant. Particularly, chromothripsis SVs was significantly associated with CNVs. Furthermore, mutational landscape of different subtypes indicated differences between subtypes, and there seems to be more commonalty between our cohort and SCLC than with other subtypes. *SMARCA4* mutations may be specific driver gene alteration in our cohort.

**Conclusion:** Our results support that LCLC and LCNEC tumors follow distinct tumorigenic pathways. To our knowledge, this is the first genome-wide profiling comparison of LCLC and LCNEC.

## Introduction

Lung cancer, commonly divided into small cell lung cancer (SCLC) and non-small cell lung cancer (NSCLC), is the leading cause of cancer-related mortality worldwide (Bray et al., 2018). Large-cell lung cancer (LCLC) is the third most common NSCLC subtype after lung adenocarcinoma (LUAD) and squamous cell carcinoma (LUSC), representing 2%–3% of NSCLC (Howlader N et al., 2015). Compared to other NSCLCs, LCLC is more malignant due to faster growth and earlier metastasis (Asamura et al., 2006; Shi et al., 2020). Histopathologically, the diagnosis of LCLC is usually excluded from LUAD, LUSC, and SCLC. LCLC is defined as an undifferentiated NSCLC without glandular or squamous cell differentiation in the WHO2004 lung cancer classification, while LCNEC was defined as LCLC with neuroendocrine morphological characteristics and at least one positive neuroendocrine immunohistochemical (IHC) marker (Travis et al., 2004). However, the 2015 WHO classification protocol (Brambilla et al., 2015) now isolates large cell neuroendocrine carcinoma (LCNEC) from LCLC tumors. Furthermore, LCLC expressing previously histologically defined lung cell markers (TTF1, Napsin A) were reclassified as LUAD, and squamous marker positive LCLC (P40, CK5/6, P63) were classified as non-keratinized or basal cell LUSC. Tumors that are surgically removed without expression of these markers are defined as LCLC.

It is of clinical importance to accurately distinguish histological subtypes. Subtype-directed diagnosis and treatment have been widely established for LUAD, LUSC, and SCLC. However, LCLC tumors are still fairly vaguely defined at the molecular level compared to other subgroups, especially given the otherwise strong molecular efforts of the 2015 WHO classification scheme. Some studies of smaller gene sets found abnormal expression of *TP53* in LCLC and LCNEC tumors, with *KRAS* mutations predominating in LCLC (Iyoda et al., 2004; Rossi et al., 2014). There was also a difference in the frequency of oncogene mutations between WHO2004 LCLC tumors expressing LUAD or LUSC markers and those with invalid markers (Rekhtman et al., 2013; Karlsson et al., 2015; Pelosi et al., 2015; Driver et al., 2016). However, studies on the genome-wide altered landscape of LCLC are lacking. In this study, we aimed to investigate the whole-genome landscape of LCLC and LCNEC tumors in relation to other histological subgroups of lung cancer. Our results link recent lung cancer classification schemes to the genome-wide landscape of the disease, supporting that LCLC and LCNEC tumors follow distinct tumorigenic pathways. To our knowledge, this is the first genome-wide profiling comparison of LCLC and LCNEC.

## Materials and methods

### Patient and tissue selection

A total of 23 LCLC and 15 LCNEC patients who have undergone surgical resection from June 2017 to December 2020 were retrospectively included and analyzed in this study, including 36 males and 2 females. All patients provided written informed consents. This study was approved by the Ethics Committee of Tongji Hospital, Tongji Medical College, Huazhong University of Science and Technology (TJ-IRB20220639). The clinical characteristics of the patients were summarized in Table 1. The diagnosis of LCLC and LCNEC were confirmed by two experienced pathologists.

**TABLE 1:** The patient characteristics and clinicopathological data.

Characteristic	LCLC*	SCLC (U Cologne)<	LUAD (TCGA)	LUSC (TCGA)
Total number	38	120	586	511
Histology				
LCLC	23 (60.5%)			
LCNEC	15 (39.5%)			
Age (y/o)				
≥60	21 (55.3%)			
<60	14 (36.8%)			
unkown	3 (7.9%)			
Gender				
male	36 (94.7%)			
female	2 (5.3%)			
Smoking				
No	9 (23.7%)			
Yes	29 (76.3%)			
Drinking				
No	23 (60.5%)			
Yes	15 (39.5%)			
Family History				
No	28 (73.7%)			
Yes	10 (26.3%)			
PD-L1 expression				
negative	12 (31.6%)			
positive	26 (68.4%)			
Tumor grade				
T1/T2	17 (44.7%)			
T3/T4	21 (55.3%)			
Lymph node metastasis				
No	25 (65.8%)			
Yes	13 (34.2%)			
Radiation/chemotherapy				
No	14 (36.8%)			
Yes	24 (63.2%)			
Patients evaluable for				
mutations	38 (100%)	120	586	511
CNV	38 (100%)			
SV	38 (100%)			

LCLC* implies WHO2004 classification.

### Immunohistochemistry

As previously described, standardized institutional protocols were used for immunohistochemical staining. The whole-slide serial tissue sections from FFPE surgical resection specimens were used to determine the expression levels for PD-L1, P40, CK5/6, P63, TTF1, Napsin A, Ki-67 and other tumor biomarkers. The PD-L1 expression was evaluated by two methods, including the tumor proportion score (TPS), defined as the percentage of viable tumor cells showing partial or complete membrane staining at any intensity (A TPS≥1% was considered as positive), and combined positive score (CPS), defined as the number of PD-L1-positive cells (tumor cells, macrophages and lymphocytes) divided by the total number of tumor cells and multiplied by 100.

### Whole genome sequencing and analysis

DNA was extracted from the tumors and paired para-cancer FFPE tissues using the QIAamp DNA FFPE tissue kit (Qiagen, United States). The resultant DNA was then quality-controlled using Nanodrop and Qubit (Thermo Fisher Scientific, United States) to ensure adequate purity and quality. Illumina paired-end libraries were prepared from extracted DNA and sequenced on Illumina HiSeq platforms (Illumina, San Diego, United States), with a mean average coverage of 50 × for both tumors and matched para-cancer tissues.

Burrows-Wheeler Aligner (BWA) was used to align the Paired-end sequencing reads to the human reference genome (hg19), and GATK 4.0 was used to sort and remove PCR duplicates. Somatic single nucleotide variants (SNVs), insertions, and deletions (indel) with default parameters were called with Strelka2 with default parameters (Kim et al., 2018). The ANNOVAR was used to annotate possible variant candidates. Germline mutation was called using best practices with the Genome Analysis Toolkit (GATK) HaplotypeCaller (version 3.6) as previously described (McKenna et al., 2010).Somatic copy number variations (CNVs) identified by FACETS and recurrently occurring CNVs were detected with GISTIC2.0. The GISTIC2.0 was used to identify regions of the genome that are significantly amplified or deleted across a set of samples (Mermel et al., 2011). CNV burden was calculated based on the identified copy number variants as previously described (Wolf et al., 2019), and then the average CNV burden was estimated for each patient. Somatic structural variants were called with ShatterSeek (Cortes-Ciriano et al., 2020) and Manta (Chen et al., 2016).

### DDR gene status analysis

DDR inactivation mutation status was determined by retrieving and combining DNA data copy number variation and single nucleotide variation of DDR genes (Tian et al., 2020). Alterations in the DDR pathway were defined as any non-synonymous somatic mutation in the protein-coding region, or homozygous deletions of at least one genes in DDR-related pathway.

### Statistics

All analyses were performed using SPSS (version 25 for Windows, Armonk, NY: IBM Corp.). Patient characteristics were evaluated with descriptive statistics. Correlation of histological classification with DDR gene status, PD-L1 expression, age, gender, stage, smoking and drinking was investigated using the chi-square test. All reported *p* values were two-sided and considered statistically significant at *p* < 0.05, unless otherwise specified.

## Results

### Clinical characteristics

All participants were pathologically reviewed, and 23 LCLC patients and 15 LCNEC patients were included in the retrospective analysis. The clinical characteristics of patients are summarized in Supplementary Table S1. Twenty-six (68.4%) patients were positive and 12 patients were negative for programmed death ligand-1 (PD-L1) staining. All of the LCNEC patients were male, and 80% of them were current or former smokers. There were no significant differences in age of diagnosis, gender, smoking, drinking history, tumor stage, lymph node metastasis and treatment between LCLC and LCNEC patients (Supplementary Table S1). Interestingly, *TP53*/*RB1* co-mutations, an important molecular subtype of LCNEC, were not present in the 15 LCNEC patients in our cohort.

### Mutational landscape of LCLC and LCNEC

**FIGURE 1:**
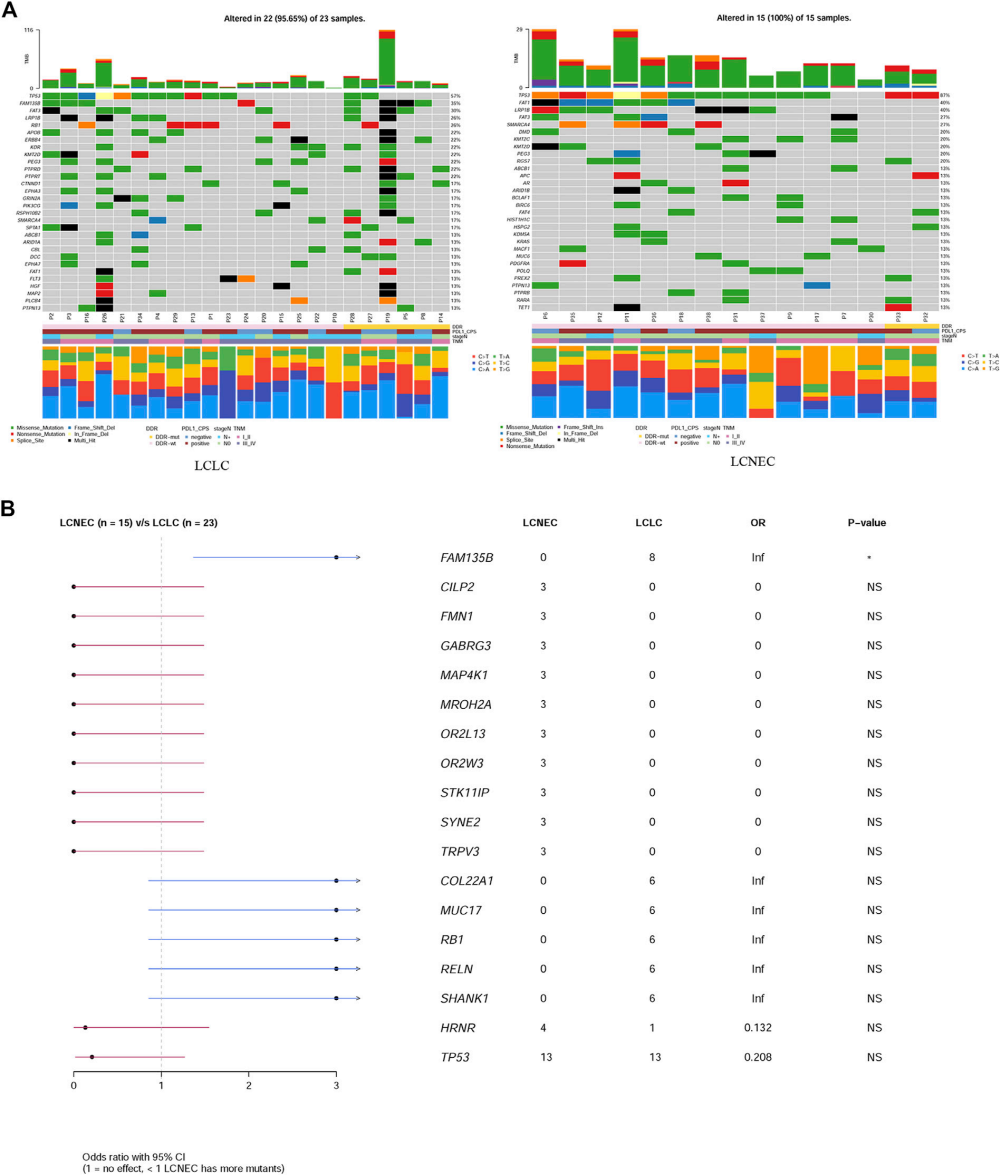
Comparison of mutation landscape between LCLC and LCNEC. **(A)** A comparison of the mutational landscapes of LCLC and LCNEC is provided, along with the most frequently mutated driver genes. The top panel represents the TMB and the middle panel represents the matrix of frequently mutated genes. Columns represent samples, and clinicopathological characteristics of individual patients are presented below. Bar plots in the lower panel shows the contribution of six substitutions. **(B)** Forestplot shows the significant differences of driver genes between the two groups.

Gene mutation profiles of 38 patients with LCLC and LCNEC were analyzed by whole genome sequencing. In total, 9931 non-synonymous somatic mutations were identified in 6013 genes. Thirty-seven of the 38 patients, including 22 LCLC patients and 15 LCNEC patients, showed at least one gene variant. *TP53* and *TTN* were the most common variants in LCLC and LCNEC (Supplementary Figure S1A). Specifically, the mutations of *CILP2*, *FMN1*, *GABRG3*, *MAP4K1*, *MROH2A*, *OR2L13*, *OR2W3*, *STK11IP* and *SYNE2* occurred only in LCNEC group (3/15, 20%, *p* = 0.054, respectively), while *FAM135B* (8/23, 35%, *p* = 0.013) mutations only occurred in LCLC group (Supplementary Figure S1B). *TP53* mutations were the dominant driver gene alteration in both LCLC (13/23, 57%) and LCNEC (13/15, 87%) tumors (*p* = 0.077) (Figure 1A). The frequency of other driver gene variants found was much lower in both subgroups. *FAM135B* (8/23, 35%) and *FAT3* (7/23, 30%) were the second and third most commonly mutated genes in LCLC, while their counterparts in LCNEC were *LRP1B* (6/15, 40%) and *FAT1* (6/15, 40%). These alterations highlight more general differences between the two subgroups. Interestingly, *RB1* (6/23, 26%, *p* = 0.063) mutations were also exclusively found in LCLC cases (Figure 1B). DDR inactivation mutation status was also identified, but in this relatively small retrospective cohort, we found no significant differences in DDR status, PD-L1 expression, lymph node metastasis, or tumor grade associated with histological classification.

**FIGURE 2:**
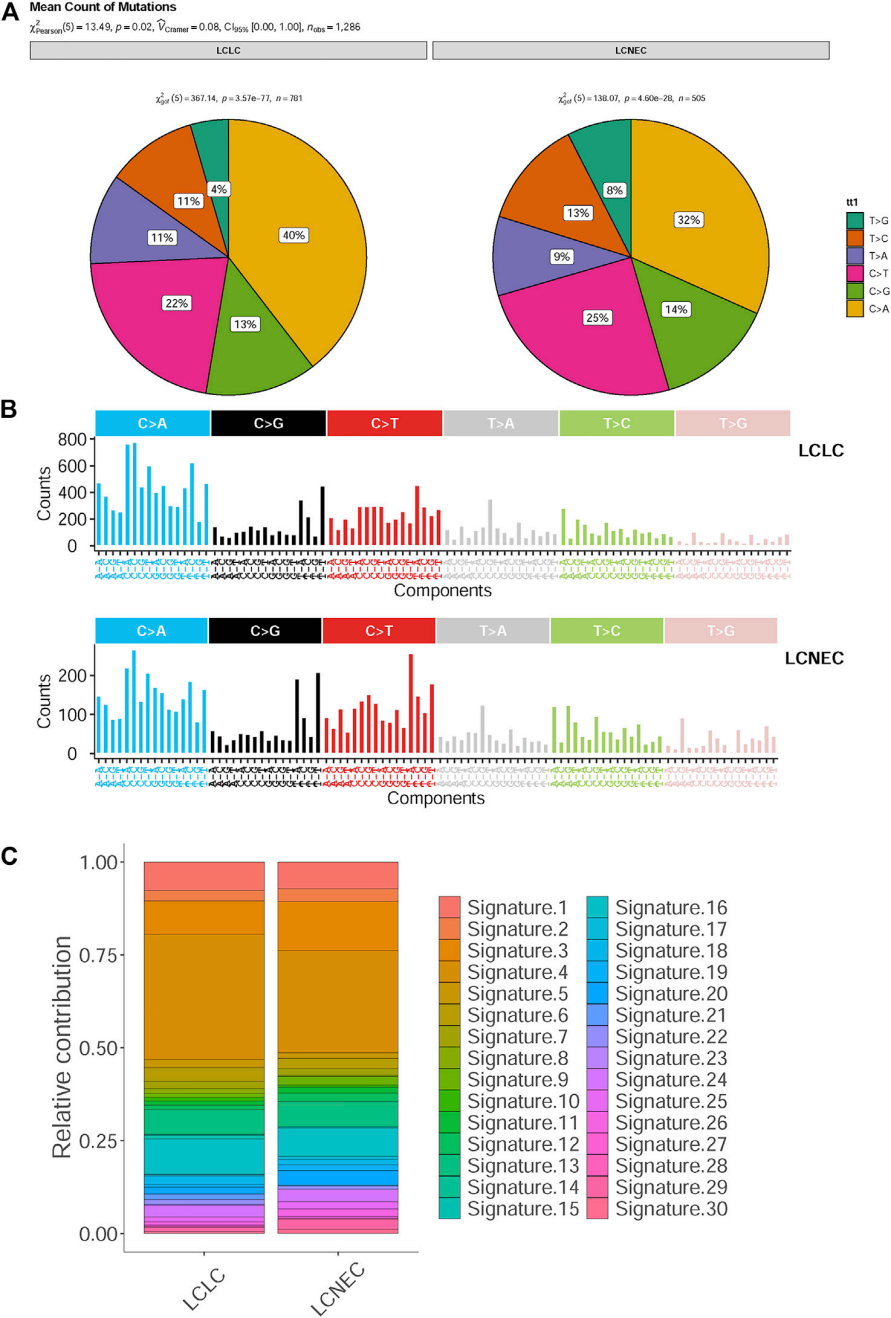
Mutational spectrum analysis for LCLC and LCNEC. **(A)** The pie chart shows the relative contribution of the six substitutions. **(B)** Relative contribution of 96 substitution subtypes SNV in each group. **(C)** Relative contributions of mutational signatures in each group.

In addition, a high frequency of C > A with accompanying C > G has been observed in both LCLC and LCNEC (Figure 1A), indicating a signature of tobacco exposure. To determine the association between the distribution of mutations and cosmic signatures in LCLC and LCNEC patients, mutation signature analyses were performed for all point mutations and the surrounding trinucleotide context. Mutational spectrum of six substitutions revealed a high frequency of C>A transversions and C>T transitions in LCLC and LCNEC. The median percentages of variants of C > A, C > G, C > T, T > A, T > C, and T > G were −40%, 13%, 22%, 11%, 11%, and 4% respectively in LCLC, while −32%, 14%, 25%, 9%, 13%, and 8% respectively in LCNEC (*p* = 0.02) (Figure 2A; Supplementary Figure 2A **)**. The profiles of 96 substitutions exhibited similar results (Figure 2B; Supplementary Figure 2B **)**.

Based on the proportion of mutation signatures in each sample and unsupervised hierarchical clustering, the patients were divided into 5 clusters (Supplementary Figure S2C). The mutation patterns of our cohort were similar to the characteristics of COSMIC “signature 4,″ “signature 5” and “signature 13.“ Unsupervised similarity analysis of tumor mutation spectrum of all published signature patterns confirmed that the maximum cosine similarity with these signatures was 0.964, 0.868, and 0.848 respectively (Supplementary Figure S2D). However, there was no significant difference in cosmic signatures between LCLC and LCNEC (Figure 2C). Signatures 3 and 4 were mainly identified in both the LCLC and LCNEC groups, with signature 3 being associated with deficiencies in DNA-double-strand break repair and signature 4 being linked to tobacco-induced mutations. Additionally, the differences between groups were analyzed according to the cluster groups, such as age, drinking, gender, smoking, grade and histopathology, but the results were not statistically significant (Supplementary Figure S2E). Moreover, the tumor mutational burden (TMB) of clustering samples (cluster 2) related to “signature 4” was generally higher (Supplementary Figure S3A). We also found that the mean value of TMB in the LCLC and LCNEC groups was 6.62 mutations per million base pairs (MB) and 4.16 mutations/MB, but the differences were not significant (*p* = 0.2) (Supplementary Figure S3B). The average weighted Genome Instability Index (wGII) score was 0.254 in the LCLC group and 0.309 in the LCNEC group, with no significant difference (*p* = 0.65) (Supplementary Figure S3C).

### CNV profiles of LCLC and LCNEC

**FIGURE 3:**
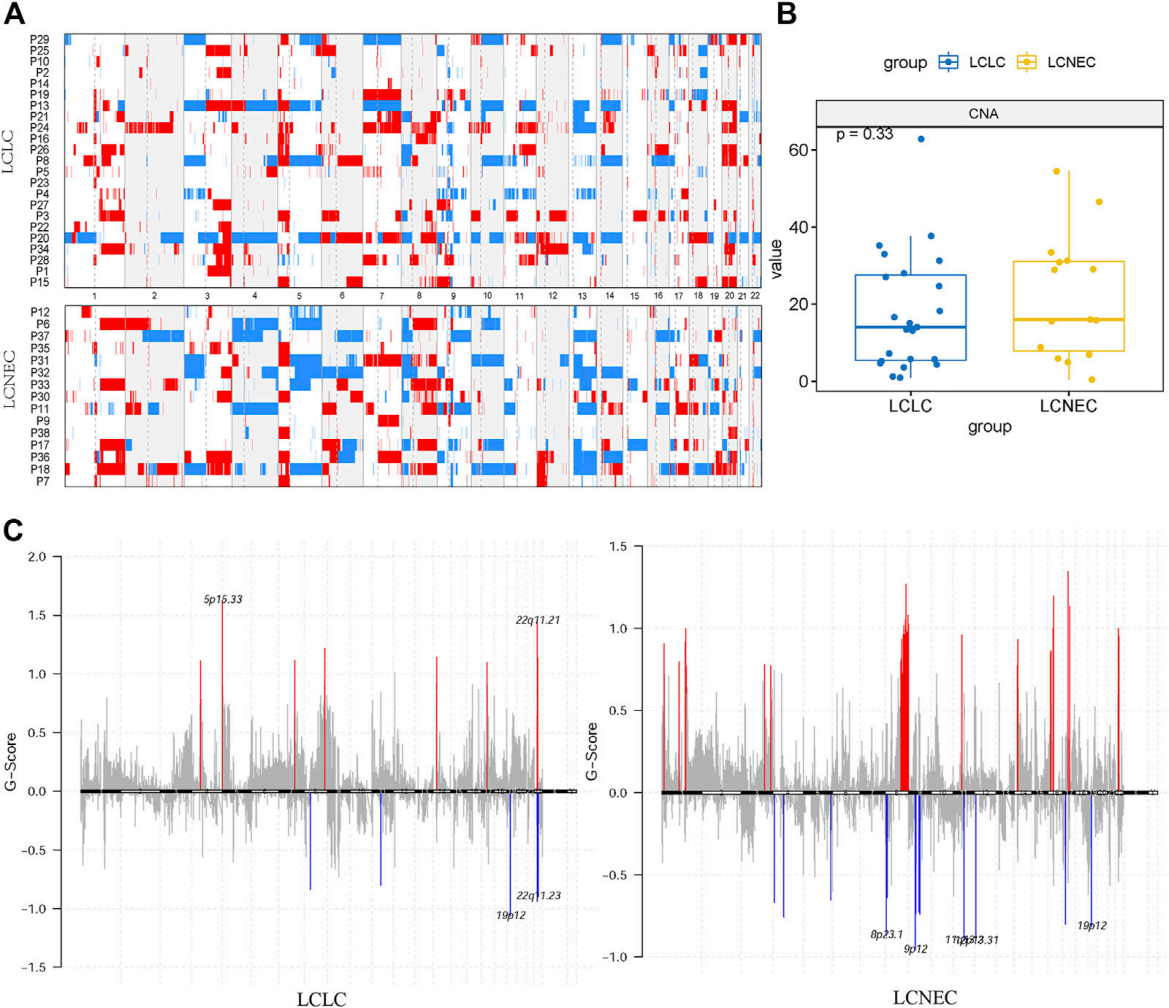
Distinct CNA landscape of LCLC and LCNEC. **(A)** Overall copy number variation (CNV) profile of LCLC and LCNEC. Red represented amplification and blue represented deletion. **(B)** Comparison of the CNA burden between LCLC and LCNEC. **(C)** Somatic copy number alterations in each group. Deletions and amplifications are represented on the y-axis by blue or red bars, respectively. Each peak region (cytoband) is displayed together with its known or potential cancer-related genes.

To characterize specific copy number variations (CNVs), we identified differential copy number variation genes between LCLC and LCNEC groups (Figure 3A). The LCNEC group exhibited a higher rate of chromosome CNV compared with the LCLC group, corresponding to higher CNV burden (22.87/case *vs* 17.15/case), but the difference was not significant (*p* = 0.33) (Figure 3B). GISTIC2.0 was used to identify significantly amplified or deleted regions of the genome across a set of samples. CNVs were found throughout the genome, with copy number gains being more prevalent than copy number losses. Chromosomes 12p13.31, 19p12, and 9q21.11 were lost and chromosomes 8q24.21, 14q11.2, 16p11.2, and 17q12 were amplified in both LCLC and LCNEC groups. Some chromosomes with CNVs were only identified in LCLC, such as 5P15.33, 7q22.1, and 22q11.23, while some were only identified in LCNEC, such as 9p12, 11q13.2, 8p23.1, 17p11.2, 4q13.2, and 9q12 (Figure 3C, Supplementary Table S2). Specifically, the copy number gains were found at chromosomes 11q11, 17q21.31, 8p11.22, and 1p36.21 in LCNEC, while the copy number losses occurred at these chromosomes in LCLC.

### SV patterns of LCLC and LCNEC

**FIGURE 4:**
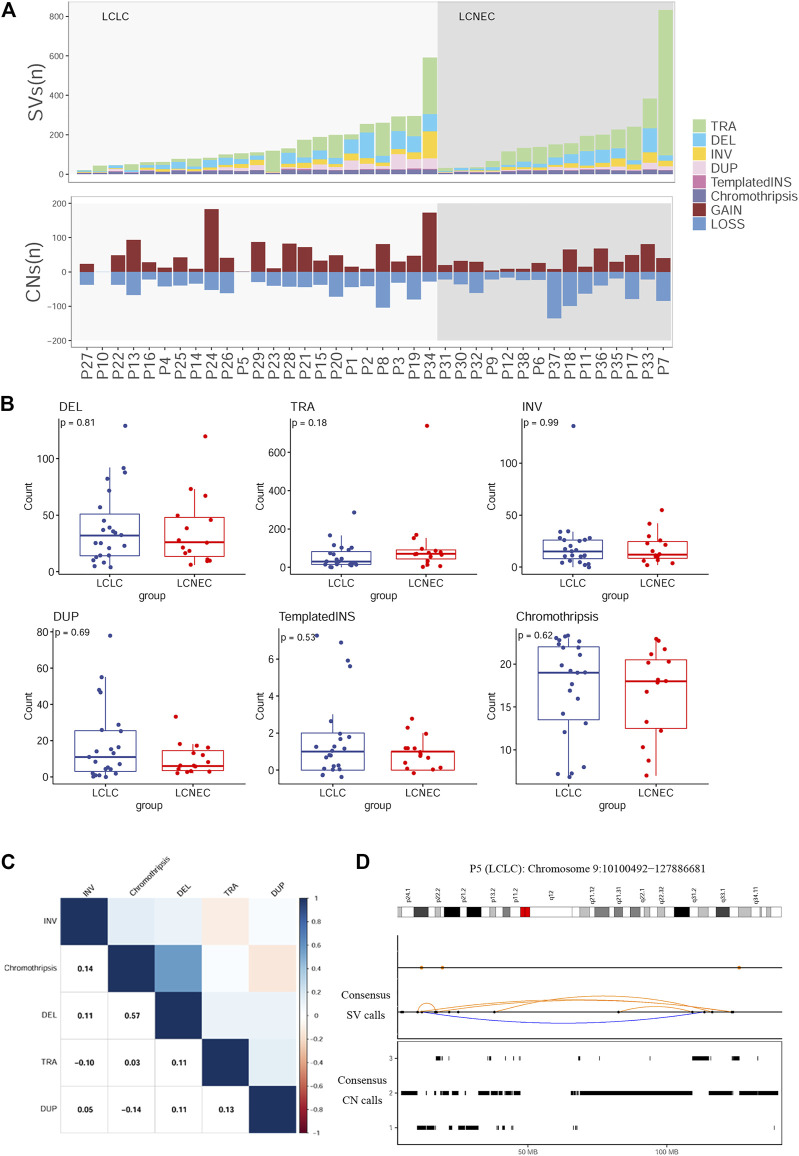
Distribution of SVs in LCLC and LCNEC **(A)** Stacked bars show the genome-wide burden of each SV class (color) in each patient (x-axis). Lower panel: SVs resulting in copy-number gain or loss **(B)** Comparison of each SV burden between LCLC and LCNEC. **(C)** Pairwise associations between the numbers of SVs across patients. Color was determined by the magnitude of positive (blue) and negative (red) Spearman correlation coefficients, plotted only where q < 0.1. **(D)** Example of a chromothripsis event in chromosome 9 involving CN oscillations with interspersed loss of heterozygosity and templated insertions. Breakpoints corresponding to interchromosomal SVs are depicted as colored dots in the SV profile, whereas intrachromosomal SVs are represented with black dots and colored arcs.

To define the patterns of structural variation (SV), ShatterSeek (Cortes-Ciriano et al., 2020) and Manta (Chen et al., 2016) were integrated to implement our final SV catalog. We identified a median of 111 SVs per LCLC patient (range 21–591) and 151 SVs per LCNEC patient (range 31–833) (Figure 4A). Translocation (TRA) accounted for the greatest proportion of all categories (47%), followed by deletion (DEL) at 22%.The count of each SV class was not significantly different between LCLC and LCNEC patients (Figure 4B). In all patients, the different SV classes showed clear patterns of co-occurrence, mutual exclusion, and association with recurrent molecular alterations. For example, the burden of chromothripsis SVs per patient was significantly positively correlated with the number of single deletions (Spearman *p* = 0.57), and negative correlation with single tandem duplication (DUP) (Figure 4C). Furthermore, our results suggest that chromothripsis SVs may be significantly associated with CNVs. We present example of a chromothripsis event in chromosome 9 with CN oscillations that span 3 CN levels showing interspersed loss of heterozygosity and templated insertions, as evidenced by their size, and breakpoint orientations at their edges (Figure 4D). Similar results were also presented in the chromosomes of LCNEC patients as illustrated in Supplementary Figure S4A.

### Comparative analysis with other pathological subtypes

**FIGURE 5:**
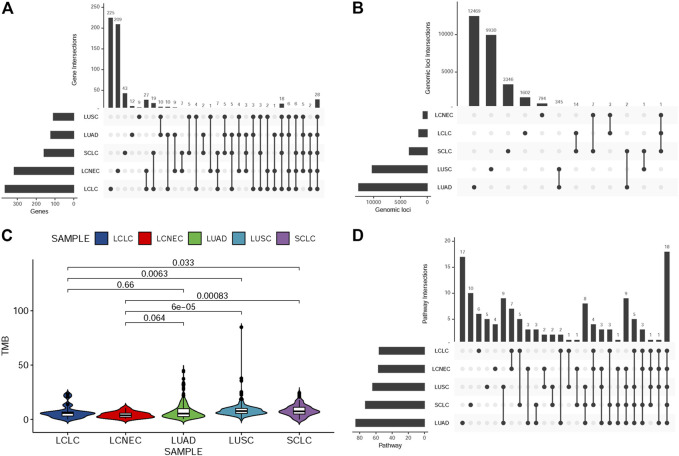
Comparison of mutation landscape between this cohort and other three subtypes. **(A)**UpSetR plot shows the overlap of germline-regulated genes identified in the present study for the five lung cancer subtypes. **(B)** UpSetR plot shows the overlap of independent genomic loci that represent the genes shown in **(A)**. **(C)** The TMB difference in the five lung cancer subtypes. **(D)**UpSetR plot shows the overlap of pathways from the Kyoto Encyclopedia of Genes and Genomes enriched with the germline-regulated genes.

To explore the unique driver genes in LCLC, we compared typical somatic mutation profiles with other lung cancer subtypes (586 LUADs and 511 LUSCs in TCGA and 120 SCLCs in U Cologne). All subtypes share 28 genes and these genes are located in different regions of the genome (FIGURE 5A,B). In addition, we compared the mutation frequency of TOP20 driver genes with other lung cancer subtypes. Results showed that most of these genes had no significant difference in mutation frequency between LCLC* and SCLC (Table 2). Notably, the mutation frequencies of *TP53*, *SMARCA4*, and *RB1* differed significantly across subtypes, suggesting that these may be specific driver gene characteristics of LCLC*, especially *SMARCA4*. Furthermore, the mutation sites of *TP53* and *RB1* were compared with those of the other three subtypes. Most mutation sites of *TP53* are located in the *P53* DNA-bingding domain. Meanwhile, the same *RB1* mutation was found only in SCLC (Table 3, Supplementary FigureS4B). Regarding the TMB, we found significant differences between LCLC and LUSC (*p* = 0.0063) or SCLC (*p* = 0.033), but not between LCLC and LUAD, respectively (Figure 5C). To gain a deeper understanding of the biological characteristics driven by these germline regulatory genes, we performed a biopathway enrichment analysis of genes in each subtype. WebGestalt was used to identify pathways that were significantly enriched in each subtype using the Kyoto Encyclopedia of Genes and Genomes (KEGG) pathway database. We found that all subtypes share 18 pathways, including small cell lung cancer pathways and non-small cell lung cancer pathways (Figure 5D).

**TABLE 2:** Comparison of TOP20 driver genes in different lung cancer subtypes.

	LCLC* (n = 38)	LUAD (n = 586)	*p*-value	LUSC(n = 511)	*p*-value	SCLC (n = 120)	*p*-value
**TP53**	68.42%	111 (21.51%)	<0.001	146 (29.08%)	<0.001	103 (85.83%)	0.0282
LRP1B	31.58%	88 (17.05%)	0.0457	153 (30.48%)	0.8571	51 (42.50%)	0.2587
FAT3	28.95%	50 (9.69%)	0.0013	32 (6.37%)	<0.001	22 (18.33%)	0.1741
FAT1	23.68%	40 (7.75%)	0.0035	55 (10.96%)	0.0325	16 (13.33%)	0.1339
FAM135B	21.05%	64 (12.40%)	0.1339	70 (13.94%)	0.2324	26 (21.67%)	1
KMT2D	21.05%	22 (4.26%)	0.0004	40 (7.97%)	0.0133	22 (18.33%)	0.8126
PEG3	21.05%	26 (5.04%)	0.0011	35 (6.97%)	0.0067	14 (11.67%)	0.1785
**SMARCA4**	21.05%	22 (4.26%)	0.0004	18 (3.59%)	0.0002	5 (4.17%)	0.0029
ERBB4	15.79%	26 (5.04%)	0.0167	33 (6.57%)	0.0468	10 (8.33%)	0.2179
PTPRT	15.79%	33 (6.40%)	0.0420	33 (6.57%)	0.0468	11 (9.17%)	0.2448
**RB1**	15.79%	23 (4.46%)	0.0102	33 (6.57%)	0.0468	87 (72.50%)	<0.001
ABCB1	13.16%	25 (4.84%)	0.0465	34 (6.77%)	0.1810	7 (5.83%)	0.1620
APOB	13.16%	43 (8.33%)	0.3621	36 (7.17%)	0.1958	16 (13.33%)	1
DMD	13.16%	45 (8.72%)	0.3734	66 (13.15%)	1	21 (17.50%)	0.6229
EPHA3	13.16%	29 (5.62%)	0.0740	38 (7.57%)	0.2132	3 (2.50%)	0.0202
KDR	13.16%	29 (5.62%)	0.0740	41 (8.17%)	0.3579	6 (5.00%)	0.1354
KMT2C	13.16%	57 (11.05%)	0.6007	37 (7.37%)	0.2042	12 (10.00%)	0.5587
PTPN13	13.16%	9 (1.74%)	0.0015	18 (3.59%)	0.0174	6 (5.00%)	0.1354
PTPRD	13.16%	91 (17.64%)	0.6570	68 (13.55%)	1	12 (10.00%)	0.5587
SPTA1	13.16%	100 (19.38%)	0.5187	60 (11.95%)	0.7962	23 (19.17%)	0.4729

LCLC* implies WHO2004 classification.

**TABLE 3:** Distribution of mutations in different subtypes of lung cancer.

	LCLC* (n = 38)	LUAD (n = 586)	LUSC (n = 511)	SCLC (n = 120)
TP53	R280G (n = 1, 3%)	R280G (n = 1, 0.2%)	0	0
	R158L (n = 1, 3%)	R158L (n = 3, 0.5%)	0	0
	E271* (n = 1, 3%)	0	E271* (n = 1, 0.2%)	0
	R248W (n = 1, 3%)	0	R248W (n = 1, 0.2%)	0
	R249S (n = 1, 3%)	0	R249S (n = 1, 0.2%)	0
	X307_splice (n = 1, 3%)	0	X307_splice (n = 1, 0.2%)	0
	M237I (n = 1, 3%)	M237I (n = 1, 0.2%)	M237I (n = 1, 0.2%)	0
	R248L (n = 1, 3%)	R248L (n = 1, 0.2%)	R248L (n = 1, 0.2%)	0
	R283P (n = 1, 3%)	R283P (n = 1, 0.2%)	R283P (n = 1, 0.2%)	0
	X225_splice (n = 1, 3%)	X225_splice (n = 1, 0.2%)	X225_splice (n = 1, 0.2%)	0
	R181P (n = 1, 3%)	0	0	R181P (n = 1, 0.8%)
	E294* (n = 1, 5%)	0	0	E294* (n = 1, 0.8%)
	R158P (n = 1, 3%)	R158P (n = 1, 0.2%)	0	R158P (n = 1, 0.8%)
	V172F (n = 1, 3%)	0	V172F (n = 1, 0.2%)	V172F (n = 1, 0.8%)
	X125_splice (n = 1, 3%)	X125_splice (n = 1, 0.2%)	X125_splice (n = 2, 0.4%)	X125_splice (n = 1, 0.8%)
	E298* (n = 1, 3%)	E298* (n = 1, 0.2%)	E298* (n = 3, 0.6%)	E298* (n = 2, 1.7%)
				
RB1	R445* (n = 1, 3%)	0	0	R445* (n = 1, 0.8%)
	X702_splice (n = 1, 3%)	0	0	X702_splice (n = 1, 0.8%)

LCLC* implies WHO2004 classification.

## Discussion

**FIGURE 6:**
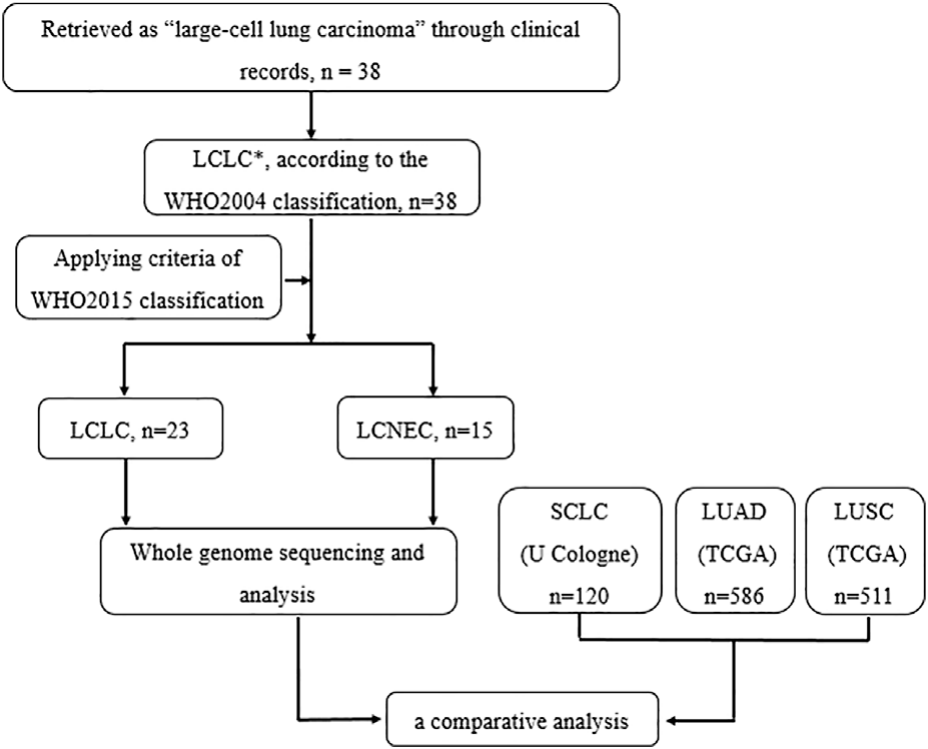
Flowchart illustrating patient enrollment and analysis.

Diagnostic terms for LCLC have been applied inconsistently in the clinic, based solely on morphology and insufficient IHC markers. It is of great importance to generate more knowledge regarding the genetic alterations in LCLC to propose more effective diagnosis and new molecular markers of predisposition and prognosis. Studies on LCLC gene profiles mainly focus on small gene sets (Karlsson et al., 2015; Pelosi et al., 2015; Driver et al., 2016), and there are few literatures on whole-genome sequencing profiling. In this study, we performed a genome-wide analysis of 23 LCLC patients and 15 LCNEC patients with a comparative analysis based on the histological classification (Figure 6). To our knowledge, this is the first genome-wide profiling comparison of LCLC and LCNEC.

The role of genes in the therapeutic efficacy of LCLC is often limited. Therefore, in the current NCCN guidelines for NSCLC, LCLC is classified as adenocarcinoma for treatment and molecular detection. In the study, frequent mutations of *TP53* were predictably observed in both LCLC and LCNEC, as well as low frequency alterations in *EGFR*, *BRAF*, and *PIK3CA* genes, consistent with previous studies (Rekhtman et al., 2013; Rossi et al., 2014). Similarly, alterations in tumor suppressors *PTEN* and *STK11* are mainly observed together with *TP53* mutations in both LCLC and LCNEC. Compared with the literature, our cohort also showed differences. We did not observe *KRAS* mutations in LCLC patients, which is the most commonly reported mutation (Karlsson et al., 2015) and this difference may be explained by ethnic differences and limited cohort size. Studies have shown that LCNEC can be further divided into SCLC-like with *TP53*/*RB1* inactivation and NSCLC-like with retained *TP53*/*RB1* functions, with different chemotherapy treatment results (Derks et al., 2018). In our study, all *RB1* mutations occurred in LCLC rather than LCNEC, and 83% (5/6) of them were co-occurring with *TP53* mutations. Unfortunately, our cohort is insufficient for further classification. Additionally, the most common mutations were found in *TTN* and *TP53* genes in both LCLC and LCNEC, with a high total frequency (45%) of a *TTN*/*TP53* double mutation. It has been suggested that *TTN* mutation or *TTN*/*TP53* co-mutation is associated with the prognosis of LUSC (Cheng et al., 2019). However, whether *TTN* is involved in lung cancer development is controversial. The focus of controversy lies in its large and complex structure and the false positive results caused by the heterogeneity of the mutation process (Hofree et al., 2013; Kim et al., 2017). Interestingly, in our cohort, *FAM135B* mutations occurred only in LCLC, with a 35% mutation rate, suggesting that the mutation may be specific to LCLC compared to LCNEC. It has been reported to have high mutation rates in other lung cancers such as LUSC (Xie et al., 2021) and SCLC (Hu et al., 2019; Wang et al., 2019). Esophageal squamous cell carcinoma has been shown to strongly express *FAM135B* with poor prognosis and silencing *FAM135B* increases radiosensitivity (Bi et al., 2021; Dong et al., 2021), but there is little evidence to support mutation as the underlying cause of elevated expression. In addition, our findings also showed differences in substitutions, copy number variations and structural variations between LCLC and LCNEC. Together, these results support that LCLC and LCNEC tumors follow different tumorigenic paths.

Furthermore, we performed a comparative analysis on the mutational profiles of histologically classified primary LUAD, LUSC, SCLC and above two subtypes. To the best of our knowledge, this is also the first study to compare and contrast these five subtypes. We used a range of different regulatory data to identify SNPs within regulatory regions of the genome that have a defined target gene. We found some overlap in SNPS, genes, and pathways among the five subtypes. LCLC and LCNEC had more private mutated genes than the other three subtypes. It is worth emphasizing that among the 28 genes shared by the 5 lung cancer subtypes, besides *TP53* and *TTN*, some other genes have been reported to be associated with lung cancer. Low-density lipoprotein (LDL) receptor-associated protein 1B (*LRP1B*), a member of the LDL receptor family, is often inactivated in lung cancer. Single gene mutations in *LRP1B* were found to be associated with high TMB in lung cancer (Lan et al., 2019), which may be associated with favorable outcomes with immune checkpoint inhibitors (Brown et al., 2021). Similarly, *FAT3* mutations have been reported to be associated with NSCLC prognosis and elevated TMB levels (Qiu et al., 2020). Interestingly, LUAD subsets with co-mutations in *FAT3* and *LRP1B* showed significantly prolonged immunotherapy progression-free survival (PFS) (Zhu et al., 2021). Mutations in anti-matrix metalloproteinase mucin 16 (*MUC16*) have been reported to be potentially associated with air pollution, thus contributing to the development of air pollution-associated lung cancer (Chen et al., 2019). *MUC16* overexpression induced by gene mutations promotes lung cancer cell growth, metastasis and chemoresistance (Lakshmanan et al., 2017; Kanwal et al., 2018). In addition to the overlapping genes mentioned above, our pathway enrichment analysis revealed that 18 biological pathways were shared among the 5 subtypes. Most of these are cancer-related signaling pathways, including pathways in small cell lung cancer and non-small cell lung cancer. The genes were also significantly enriched in important tumor onset and metastasis pathways, such as “ECM-receptor interaction” and “Focal adhesion”.

Our study has several limitations that should be noted. First, our sample size was not very large, however, due to the low incidence of LCLC, it took us 3 years and 6 months to collect these 38 samples from June 2017 to December 2020 (a total of 76 tissues, including 23 LCLCs and 15 LCNECs), making it difficult to collect more samples in the limited time available. Non-etheless, more patients and more complete clinical data (including regular follow-up) are needed in the future to validate the results of this study. Second, our study lacked other omics analyses that may provide more molecular characteristics for LCLC and LCNEC.

In this study, we aimed to investigate the whole-genome landscape of LCLC and LCNEC tumors in relation to other histological subgroups of lung cancer. Our results link recent lung cancer classification schemes to the genome-wide landscape of the disease, supporting that LCLC and LCNEC tumors follow distinct tumorigenic pathways. To our knowledge, this is the first genome-wide profiling comparison of LCLC and LCNEC.

## Data Availability

The data presented in the study are deposited in the Genome Sequence Archive repository, accession number HRA005093.
