# Supplementary material for: Whole-genome characterization of large-cell lung carcinoma: A comparative analysis based on the histological classification
Source: Front Genet. 2023 Jan 4;13:1070048. doi: 10.3389/fgene.2022.1070048 (PMC9845284; doi:10.3389/fgene.2022.1070048)
Supplement: Supplementary file 1 [file Table1.DOCX]

**Table 1. The patient characteristics and clinicopathological data**

| Characteristic | LCLC* | SCLC  (U Cologne) | LUAD (TCGA) | LUSC (TCGA) |
| --- | --- | --- | --- | --- |
| Total number | 38 | 120 | 586 | 511 |
| Histology |  |  |  |  |
| LCLC | 23 (60.5%) |  |  |  |
| LCNEC | 15 (39.5%) |  |  |  |
| Age(y/o) |  |  |  |  |
| ≥60 | 21 (55.3%) |  |  |  |
| <60 | 14 (36.8%) |  |  |  |
| unkown | 3 (7.9%) |  |  |  |
| Gender |  |  |  |  |
| male | 36 (94.7%) |  |  |  |
| female | 2 (5.3%) |  |  |  |
| Smoking |  |  |  |  |
| No | 9 (23.7%) |  |  |  |
| Yes | 29 (76.3%) |  |  |  |
| Drinking |  |  |  |  |
| No | 23 (60.5%) |  |  |  |
| Yes | 15 (39.5%) |  |  |  |
| Family History |  |  |  |  |
| No | 28 (73.7%) |  |  |  |
| Yes | 10 (26.3%) |  |  |  |
| PD-L1 expression |  |  |  |  |
| negative | 12 (31.6%) |  |  |  |
| positive | 26 (68.4%) |  |  |  |
| Tumor grade |  |  |  |  |
| T1/T2 | 17 (44.7%) |  |  |  |
| T3/T4 | 21 (55.3%) |  |  |  |
| Lymph node metastasis |  |  |  |  |
| No | 25 (65.8%) |  |  |  |
| Yes | 13 (34.2%) |  |  |  |
| Radiation/chemotherapy |  |  |  |  |
| No | 14 (36.8%) |  |  |  |
| Yes | 24 (63.2%) |  |  |  |
| Patients evaluable for |  |  |  |  |
| mutations | 38 (100%) | 120 | 586 | 511 |
| CNV | 38 (100%) |  |  |  |
| SV | 38 (100%) |  |  |  |

LCLC* implies WHO2004 classification.
