# Supplementary material for: Whole-genome characterization of large-cell lung carcinoma: A comparative analysis based on the histological classification
Source: Front Genet. 2023 Jan 4;13:1070048. doi: 10.3389/fgene.2022.1070048 (PMC9845284; doi:10.3389/fgene.2022.1070048)
Supplement: Supplementary file 2 [file Table2.DOCX]

**Table 2. Comparison of TOP20 driver genes in different lung cancer subtypes.**

|  | **LCLC* (n=38)** | **LUAD (n=586)** | **p-value** | **LUSC(n=511)** | **p-value** | **SCLC(n=120)** | **p-value** |
| --- | --- | --- | --- | --- | --- | --- | --- |
| **TP53** | 68.42% | 111 (21.51%) | <0.001 | 146 (29.08%) | <0.001 | 103 (85.83%) | 0.0282 |
| LRP1B | 31.58% | 88 (17.05%) | 0.0457 | 153 (30.48%) | 0.8571 | 51 (42.50%) | 0.2587 |
| FAT3 | 28.95% | 50 (9.69%) | 0.0013 | 32 (6.37%) | <0.001 | 22 (18.33%) | 0.1741 |
| FAT1 | 23.68% | 40 (7.75%) | 0.0035 | 55 (10.96%) | 0.0325 | 16 (13.33%) | 0.1339 |
| FAM135B | 21.05% | 64 (12.40%) | 0.1339 | 70 (13.94%) | 0.2324 | 26 (21.67%) | 1 |
| KMT2D | 21.05% | 22 (4.26%) | 0.0004 | 40 (7.97%) | 0.0133 | 22 (18.33%) | 0.8126 |
| PEG3 | 21.05% | 26 (5.04%) | 0.0011 | 35 (6.97%) | 0.0067 | 14 (11.67%) | 0.1785 |
| **SMARCA4** | 21.05% | 22 (4.26%) | 0.0004 | 18 (3.59%) | 0.0002 | 5 (4.17%) | 0.0029 |
| ERBB4 | 15.79% | 26 (5.04%) | 0.0167 | 33 (6.57%) | 0.0468 | 10 (8.33%) | 0.2179 |
| PTPRT | 15.79% | 33 (6.40%) | 0.0420 | 33 (6.57%) | 0.0468 | 11 (9.17%) | 0.2448 |
| **RB1** | 15.79% | 23 (4.46%) | 0.0102 | 33 (6.57%) | 0.0468 | 87 (72.50%) | <0.001 |
| ABCB1 | 13.16% | 25 (4.84%) | 0.0465 | 34 (6.77%) | 0.1810 | 7 (5.83%) | 0.1620 |
| APOB | 13.16% | 43 (8.33%) | 0.3621 | 36 (7.17%) | 0.1958 | 16 (13.33%) | 1 |
| DMD | 13.16% | 45 (8.72%) | 0.3734 | 66 (13.15%) | 1 | 21 (17.50%) | 0.6229 |
| EPHA3 | 13.16% | 29 (5.62%) | 0.0740 | 38 (7.57%) | 0.2132 | 3 (2.50%) | 0.0202 |
| KDR | 13.16% | 29 (5.62%) | 0.0740 | 41 (8.17%) | 0.3579 | 6 (5.00%) | 0.1354 |
| KMT2C | 13.16% | 57 (11.05%) | 0.6007 | 37 (7.37%) | 0.2042 | 12 (10.00%) | 0.5587 |
| PTPN13 | 13.16% | 9 (1.74%) | 0.0015 | 18 (3.59%) | 0.0174 | 6 (5.00%) | 0.1354 |
| PTPRD | 13.16% | 91 (17.64%) | 0.6570 | 68 (13.55%) | 1 | 12 (10.00%) | 0.5587 |
| SPTA1 | 13.16% | 100 (19.38%) | 0.5187 | 60 (11.95%) | 0.7962 | 23 (19.17%) | 0.4729 |

LCLC* implies WHO2004 classification.
