# Supplementary material for: Whole-genome characterization of large-cell lung carcinoma: A comparative analysis based on the histological classification
Source: Front Genet. 2023 Jan 4;13:1070048. doi: 10.3389/fgene.2022.1070048 (PMC9845284; doi:10.3389/fgene.2022.1070048)
Supplement: Supplementary file 3 [file Table3.DOCX]

**Table 3 Distribution of mutations in different subtypes of lung cancer.**

|  | LCLC* (n=38) | LUAD (n=586) | LUSC (n=511) | SCLC (n=120) |
| --- | --- | --- | --- | --- |
| TP53 | R280G (n=1, 3%) | R280G (n=1, 0.2%) | 0 | 0 |
|  | R158L (n=1, 3%) | R158L (n=3, 0.5%) | 0 | 0 |
|  | E271* (n=1, 3%) | 0 | E271* (n=1, 0.2%) | 0 |
|  | R248W (n=1, 3%) | 0 | R248W (n=1, 0.2%) | 0 |
|  | R249S (n=1, 3%) | 0 | R249S (n=1, 0.2%) | 0 |
|  | X307_splice (n=1, 3%) | 0 | X307_splice (n=1, 0.2%) | 0 |
|  | M237I (n=1, 3%) | M237I (n=1, 0.2%) | M237I (n=1, 0.2%) | 0 |
|  | R248L (n=1, 3%) | R248L (n=1, 0.2%) | R248L (n=1, 0.2%) | 0 |
|  | R283P (n=1, 3%) | R283P (n=1, 0.2%) | R283P (n=1, 0.2%) | 0 |
|  | X225_splice (n=1, 3%) | X225_splice (n=1, 0.2%) | X225_splice (n=1, 0.2%) | 0 |
|  | R181P (n=1, 3%) | 0 | 0 | R181P (n=1, 0.8%) |
|  | E294* (n=1, 5%) | 0 | 0 | E294* (n=1, 0.8%) |
|  | R158P (n=1, 3%) | R158P (n=1, 0.2%) | 0 | R158P (n=1, 0.8%) |
|  | V172F (n=1, 3%) | 0 | V172F (n=1, 0.2%) | V172F (n=1, 0.8%) |
|  | X125_splice (n=1, 3%) | X125_splice (n=1, 0.2%) | X125_splice (n=2, 0.4%) | X125_splice (n=1, 0.8%) |
|  | E298* (n=1, 3%) | E298* (n=1, 0.2%) | E298* (n=3, 0.6%) | E298* (n=2, 1.7%) |
|  |  |  |  |  |
| RB1 | R445* (n=1, 3%) | 0 | 0 | R445* (n=1, 0.8%) |
|  | X702_splice (n=1, 3%) | 0 | 0 | X702_splice (n=1, 0.8%) |

LCLC* implies WHO2004 classification
