# Supplementary material for: Whole-genome characterization of large-cell lung carcinoma: A comparative analysis based on the histological classification
Source: Front Genet. 2023 Jan 4;13:1070048. doi: 10.3389/fgene.2022.1070048 (PMC9845284; doi:10.3389/fgene.2022.1070048)
Supplement: Supplementary file 4 [file Table4.DOCX]

**Figure S1. Comparison of mutation landscape between LCLC and LCNEC. (A)** A comparison of the mutational landscapes of LCLC and LCNEC is provided, along with the most frequently mutated genes. The top panel represents the TMB and the middle panel represents the matrix of frequently mutated genes. Columns represent samples, and clinicopathological characteristics of individual patients are presented below. Bar plots in the lower panel shows the contribution of six substitutions. **(B)** List of genes with significant differences between the two groups.


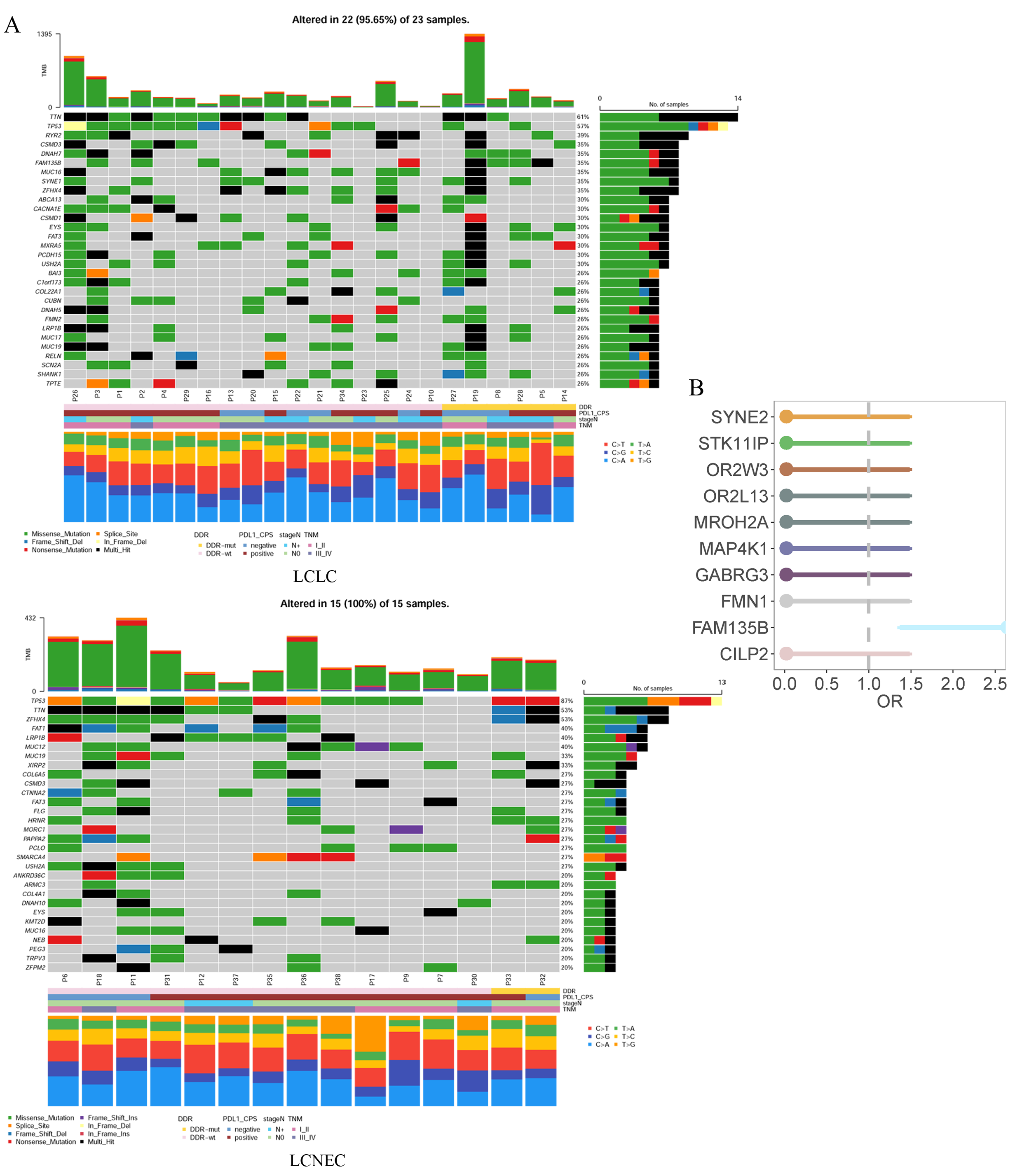


**Figure 2. Mutational spectrum analysis for LCLC and LCNEC.** **(A)** Bar plots showing the frequency of six substitutions. **(B)** The pie chart shows the relative contribution of the 96 substitutions. **(C)** The unsupervised hierarchical cluster method was applied to divide the 38 tumor samples into five clusters. **(D)** Unsupervised similarity analysis of tumor mutation spectrum of all published signature patterns. **(E)** Comparisons of clinical features of patients among the five clusters based on the Kruskal- Wallis test.


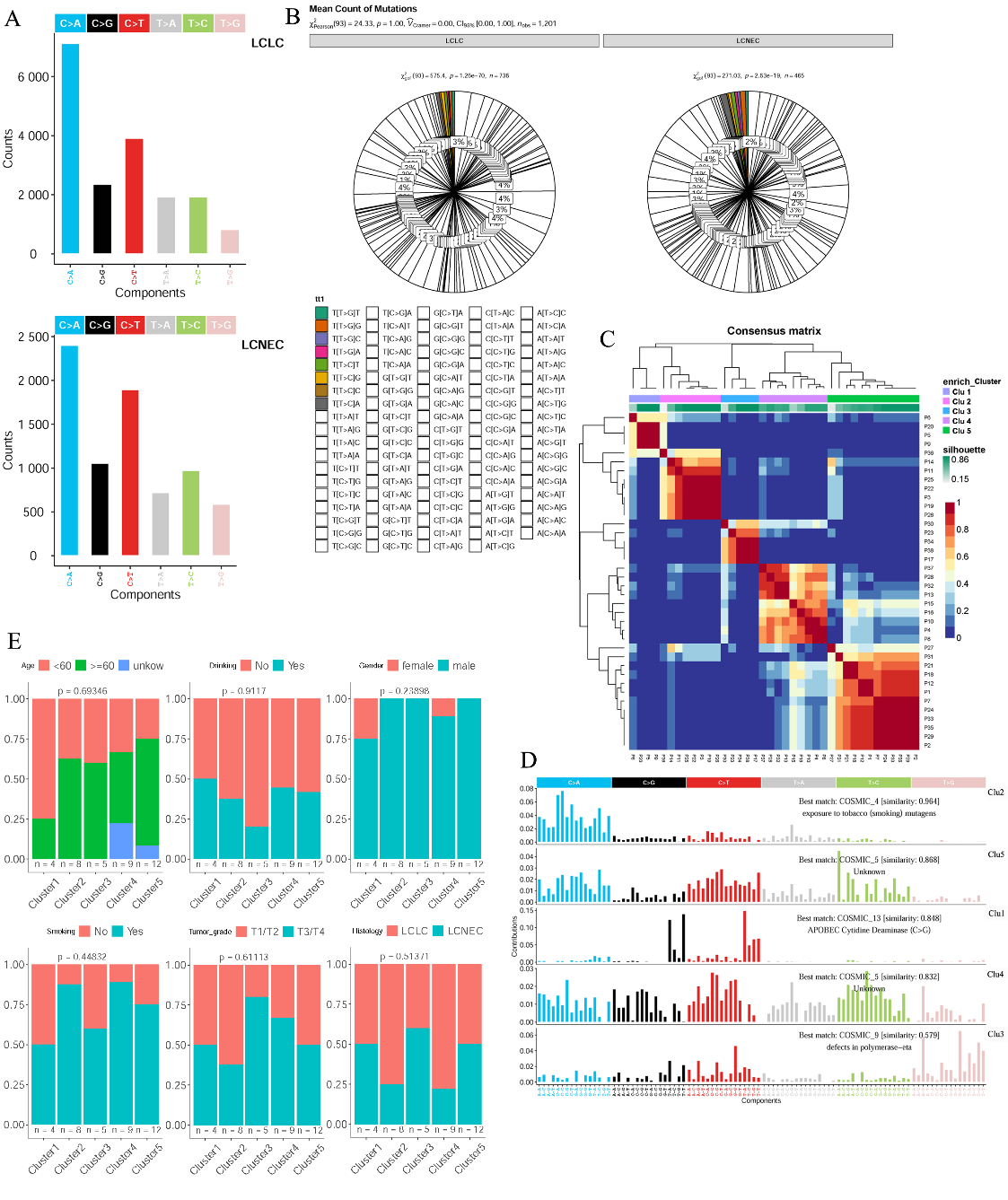


**Figure S3. TMB and wGII in LCLC and LCNEC. (A)** Comparison of TMB corresponding to 5 clusters. **(B)** The TMB difference between LCLC and LCNEC. **(C)** Comparison of wGII between LCLC and LCNEC.


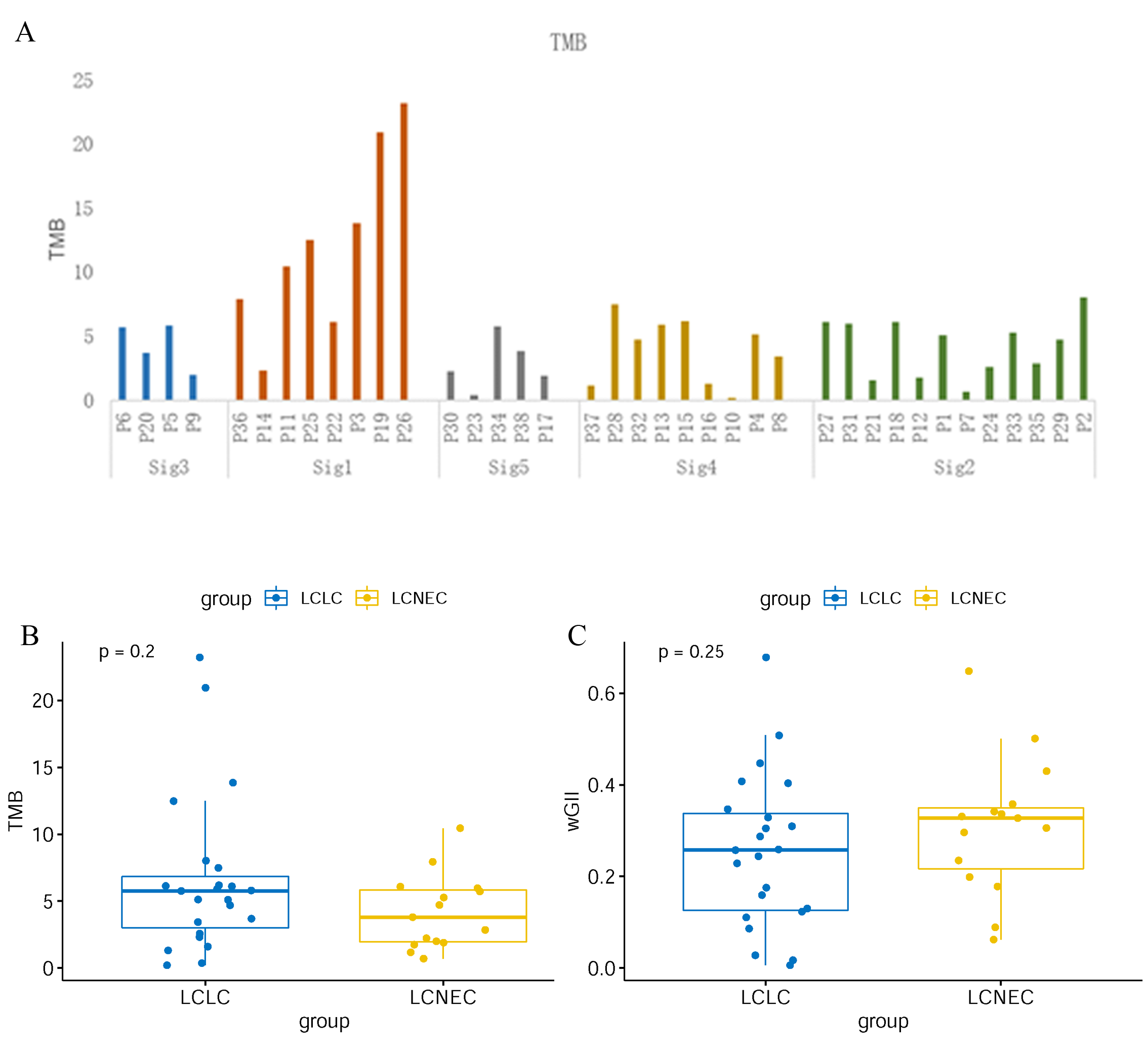


**Figure S4. Comparison of mutation landscape between this cohort and other three subtypes. (A)** Example of a chromothripsis event in the chromosomes of LCNEC patients with CN oscillations. **(B)** Comparison of TP53 and RB1 gene mutation sites in the present study with other three lung cancer subtypes.


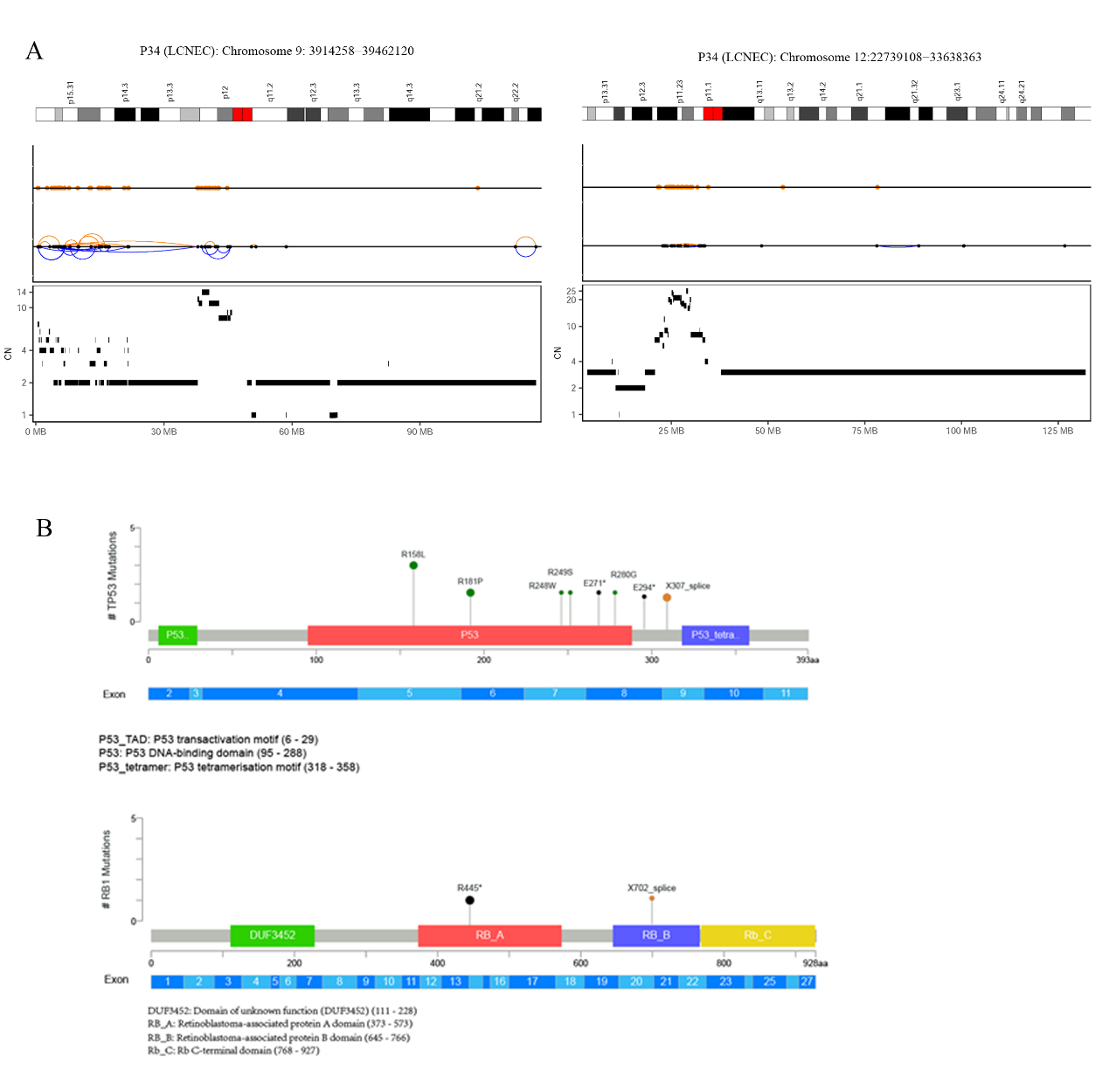


**Table S1. The clinical characteristics of the patients**

| Characteristic | LCLC(n=23) | LCNEC(n=15) | P value |
| --- | --- | --- | --- |
| Age(y/o) |  |  | 0.937 |
| ≥60 | 13 (57%) | 8 (53%) |  |
| <60 | 8 (35%) | 6 (40%) |  |
| unkown | 2 (8%) | 1 (7%) |  |
| Gender |  |  | 0.241 |
| male | 21 (91%) | 15 (100%) |  |
| female | 2 (9%) | 0 (0%) |  |
| Smoking |  |  | 0.666 |
| No | 6 (26%) | 3 (20%) |  |
| Yes | 17 (74%) | 12 (80%) |  |
| Drinking |  |  | 0.957 |
| No | 14 (61%) | 9 (60%) |  |
| Yes | 9 (39%) | 6 (40%) |  |
| Family History |  |  | 0.142 |
| No | 15 (65%) | 13 (87%) |  |
| Yes | 8 (35%) | 2 (13%) |  |
| PD-L1 expression |  |  | 0.599 |
| negative | 8 (35%) | 4 (27%) |  |
| positive | 15 (65%) | 11 (73%) |  |
| Tumor grade |  |  | 0.389 |
| T1/T2 | 9 (39%) | 8 (53%) |  |
| T3/T4 | 14 (61%) | 7 (47%) |  |
| Lymph node metastasis |  |  | 0.136 |
| No | 13 (57%) | 12 (80%) |  |
| Yes | 10 (43%) | 3 (20%) |  |
| Radiation/chemotherapy |  |  | 0.311 |
| No | 7 (30%) | 7 (47%) |  |
| Yes | 16 (70%) | 8 (53%) |  |
| TP53/RB1 co-mutation |  |  | 0.053 |
| No | 18 (78%) | 15 (100%) |  |
| Yes | 5 (22%) | 0 (0%) |  |
